# Influenza in Adults Seeking Care at Seven European Emergency Departments: A Prospective Active Surveillance During the 2019–2020 Influenza Season

**DOI:** 10.1111/irv.70040

**Published:** 2024-11-20

**Authors:** Sélilah Amour, Alberto Perez Rubio, Andrea Orsi, Michael Oppert, Micha Loebermann, Carlos del Pozo Vegas, Karim Tazarourte, Marion Douplat, Laurent Jacquin, Giancarlo Icardi, Jonas Walker, Andrea Glass, Joshua Nealon, Sandra S. Chaves, FERS Study Group, Hélène Bricout, Philippe Vanhems

**Affiliations:** ^1^ Service d'Hygiène, Epidémiologie, Infectiovigilance et Prévention Hospices Civils de Lyon Lyon France; ^2^ Public Health, Epidemiology and Evolutionary Ecology of Infectious Diseases (PHE3ID), Centre International de Recherche en Infectiologie (CIRI), INSERM U1111, CNRS UMR5308, ENS de Lyon Université de Lyon 1 Lyon France; ^3^ Dirección Médica Hospital Clínico Universitario de Valladolid Valladolid Spain; ^4^ Department of Health Sciences University of Genoa Genoa Italy; ^5^ Klinik für Notfall‐ und Internistische Intensivmedizin Klinikum Ernst von Bergmann Potsdam Germany; ^6^ Abteilung für Tropenmedizin und Infektionskrankheiten und Sektion Nephrologie Universitätsmedizin Rostock Rostock Germany; ^7^ Service d'Accueil des Urgences Hospices Civils de Lyon Lyon France; ^8^ Research on Healthcare Performance (RESHAPE), INSERM U1290 Université Claude Bernard Lyon 1 Lyon France; ^9^ INSERM UMR1060 (CarMeN), Ischemia‐Reperfusion Syndromes (IRIS) Université Lyon 1 Bron France; ^10^ Sanofi Vaccines Campus Sanofi Lyon Lyon France

**Keywords:** International Classification of Diseases (ICD) diagnosis, emergency department (ED), influenza, influenza‐like illness (ILI)

## Abstract

**Background:**

Influenza can be associated with nonrespiratory disease presentation, but these are less well documented due to the lack of routine testing for influenza in the healthcare system, especially if patients do not present with influenza‐like illness (ILI). We aimed to measure the proportion of influenza cases seeking care at emergency department (ED) for a nontraumatic cause, to describe their clinical presentation and their ED‐discharge diagnosis.

**Methods:**

The study was conducted at seven hospitals in France, Spain, Italy and Germany during the 2019–20 influenza season, for a period of 10 weeks. Patients (≥ 18 years) consulting for nontraumatic causes at the ED were invited to participate. Consenting patients provided upper respiratory swab samples for influenza testing by reverse transcription polymerase chain reaction. Clinical and demographic data were collected.

**Results:**

There were 8678 patients included, 50.7% were female and the median age was 57 years. Among them, 494 (5.7%) were laboratory‐confirmed influenza (LCI) cases. Nonetheless, only 24.3% of LCI cases had an ED‐discharge of influenza. Of all cases confirmed as influenza, 47.6% had a nonrespiratory discharge diagnosis, which frequency increased with age. ILI case definition from the European Centre for Disease Prevention and Control was the most frequently met among influenza cases (68.6%). Older patients (≥ 65 years) were less frequently identified based on any ILI signs/symptoms.

**Conclusion:**

Our findings indicate that the impact of influenza among patients seeking care at the ED cannot be easily assessed based on clinical presentation and medical records alone. Preventing influenza among adult population may reduce healthcare utilization.

## 1 Introduction

Influenza is a contagious respiratory viral infection that causes seasonal epidemics during winter months [1]. The intensity and the duration of influenza varies from season to season based on the dynamics of levels of immunity in the populations and type of circulating virus [2]. A study estimated annual influenza‐associated respiratory mortality per 100,000 individuals of all age to be around 4.0–8.8 worldwide and 3.1–8.0 in Europe [3]. Influenza can also cause unpredictable pandemics with important human and economic implications [4]. To support public health decisions related to influenza control and prevention, the World Health Organization (WHO) provides global standards for influenza surveillance systems [4, 5, 6], based on syndromic case definitions for influenza‐like illness (ILI) and severe acute respiratory infection (SARI) [7, 8]. Nonetheless, these surveillance case definitions focus exclusively on respiratory signs and symptoms.

Influenza can cause both respiratory and nonrespiratory illnesses. However, the contribution of influenza‐associated nonrespiratory disease presentation is less appreciated. This is partly because detection of influenza viruses in healthcare settings is low due to insufficient use of diagnostic tests as part of routine clinical care. Influenza is often perceived as a mild disease that mostly affects older adults. Vaccination programmes in European countries remain primarily focused on people with comorbidities and those older than 60 or 65 years (depending on country policy). Among older adults, despite recommendations for annual influenza vaccination, coverage remains suboptimum—below the WHO's recommended of at least 75% coverage [9].

To estimate the contribution of influenza among adults seeking care at emergency departments (EDs), we implemented a prospective surveillance aiming to test patients attended at the ED in four European countries during influenza season for influenza viruses and describe their disease presentation, with focus on those patients without typical respiratory signs and symptoms that could be missed in surveillance settings.

## 2 Methods

### 2.1 Study Design and Population

A hospital‐based prospective multicentre cross‐sectional study was conducted in seven university hospitals from four countries: France (2), Spain (2), Italy (1) and Germany (2) during the influenza season 2019–2020. National influenza surveillance data (site‐specific criteria applied) were used to signal the start of the study according to the local influenza activity, aiming for a total of 10 weeks of surveillance in early 2020. Study enrolment begun at Week 01/2020 with Italy and ended at Week 12/2020 with Germany. Inclusion criteria were (1) patients aged 18 years of age or above and (2) consulting for nontraumatic causes at the ED. Patients with traumatic causes [10] were excluded. All patients were invited to participate in the study, and those who agreed to take part received a printed study information and signed informed consent.

### 2.2 Data Collection

Consenting patients completed a questionnaire and additional clinical and demographic data were collected from electronic hospital records. The following data were analysed: sex, age, body mass index (BMI), comorbidities (chronic pulmonary disease [e.g., COPD or asthma], recent pneumonia, diabetes, liver disorder, kidney disorder, heart disease, immunocompromised, neurological or neuromuscular disease and other chronic conditions), recent hospitalization (< 30 days prior to the ED visit), current influenza vaccine, smoking status, signs and/or symptoms (fever ≥ 38°C, malaise, myalgia/arthralgia, headache, cough, sore throat and shortness of breath) present in the 10 days prior to the ED visit and ED visit data: duration of ED visit, post‐ED outcome and main discharge diagnosis after leaving ED.

### 2.3 Virological Investigations

In addition, for each study participant, nasopharyngeal or combined nasal and throat swabs were collected. Influenza A and B viruses were identified by reverse transcription polymerase chain reaction (RT‐PCR). Subtyping of influenza A‐positive samples and lineage of influenza B‐positive samples were also carried out (Table S1).

### 2.4 Clinical Diagnostic of Influenza and Variable Categories

The discharge diagnosis when leaving ED was grouped in 12 categories (Table S2). The variable influenza defined influenza‐specific diagnosis ICD‐09 codes (Codes 487 [Influenza] and 488 [Influenza due to certain identified influenza viruses] and their subcodes) or ICD‐10 codes (J09 [Influenza due to identified zoonotic or pandemic influenza virus], J10 [Influenza due to identified seasonal influenza virus] and J11 [Influenza, virus not identified] and their subcodes). The rate of ED visits attributed to influenza as a discharge diagnosis was calculated as number of ED visits with a medical diagnosis of influenza, divided by the total number of ED visits with a medical diagnosis. This ratio was multiplied by 10,000. The rate of hospitalizations for influenza among all hospitalizations following a visit to the ED was calculated as number of hospitalizations with a medical diagnosis of influenza, divided by the total number of hospitalizations with medical diagnosis. The ratio was multiplied by 1000.

### 2.5 Statistical Analysis

All sites aimed for a target enrolment of 5000 patients per country (except for France that focused on 3000 enrolment), with slightly variation on case‐finding according to operationally‐feasible sampling frames at each hospital (Table S3). A descriptive analysis reports the proportion of confirmed influenza cases and the demographic and medical features/profile of influenza PCR positive cases. Categorical variables were described with absolute and relative frequencies and compared by chi‐square test or Fisher exact test as appropriate. Continuous variables were described as mean (±standard deviation [SD]), median (interquartile range [IQR]) and compared by Mann–Whitney *U* test, Wilcoxon test or Kruskal–Wallis test. To assess the accuracy of the clinical diagnosis coded as of influenza, positive predictive value (PPV), negative predictive value (NPV), sensitivity (Se) and specificity (Sp) were calculated, using PCR test as the gold standard for documenting confirmed cases of influenza. Se was the probability that the clinical diagnostic was influenza among laboratory‐confirmed influenza (LCI) cases while Sp was the probability that the clinical diagnostic was negative among patients tested negative for influenza. Estimates were computed overall and stratified by age group (18–49, 50–64, 65–79 and ≥ 80 years), sex, presence of at least one chronic disease and influenza vaccination status. To consider that the clinical diagnosis is a good estimate of influenza, both Se and Sp must be high (> 90%). *p* values < 5% was considered significant, and all of the tests were two tailed. Stata SE Version 17 (StataCorp LP) was used for data analysis.

## 3 Results

### 3.1 Population Characteristics

A total of 8678 patients were enrolled in four countries, of which 44% (*n* = 3810) were from Spain. The median age was 57 years (range 18 to 108), and 51% (*n* = 4386) were female (Table 1). Sixty‐one per cent had at least one comorbidity, and 33% were vaccinated against influenza for the study season (Table 1). By country, influenza vaccination coverage was 29% in France, 33% in Italy, 34% in Spain and 41% in Germany (Table 1). The proportion of patients with comorbidities increased with age: 34% in the 18–49 years versus 65%, 84% and 92% in the 50–64, 65–79 and ≥ 80 years, respectively (Table S4). Similarly, influenza vaccination coverage increased significantly with age: 11% and 26% in 18–49 and 50–64 years, respectively, compared to 56% and 70% in 65–79 and ≥ 80 years, respectively (*p* < 0.001) (Table S4). Almost 5% of patients had been hospitalized in the 30 days prior to their emergency room visit with rates increasing with age: 2.5% in the 18–49 years versus 4.3%, 6.7% and 7.9% in the 50–64, 65–79 and ≥ 80 years, respectively (*p* < 0.001) (Tables 1 and S4). Influenza vaccine coverage was significantly higher among patients tested negative for influenza compared to LCI cases (35% vs. 24%, respectively, *p* < 0.001) (Table S5). Additional baseline characteristics according to LCI status are shown in Table S5.

**TABLE 1:** Baseline characteristics according to country, 2019–2020 (*n* = 8678).

	France *N* = 2478		Italy *N* = 1307		Spain *N* = 3810		Germany *N* = 1083		Total *N* = 8678	
	*N*	%	*N*	%	*N*	%	*N*	%	*N*	%
Age distribution (years)										
18–49	1172	47.3	407	31.4	1674	44.0	273	25.2	3526	40.6
50–64	426	17.2	279	21.4	764	20.0	202	18.7	1671	19.3
65–79	489	19.7	339	25.9	864	22.7	274	25.3	1966	22.7
> 80	391	15.8	282	21.6	500	13.1	316	29.2	1489	17.2
Mean age (±standard deviation [SD])	53.0 (±22.4)		60.7 ± 20.5		54.3 ± 20.9		63.7 ± 20.3		56.1 ± 21.6	
Median age (interquartile range [IQR])	51.0 (40.0)		63.4 32.8		54.3 35.3		67.0 32.0		57.0 37.4	
Range	18.0–99.0		18.0–98.1		18.0–107.6		18.0–102.0		18.0–107.6	
Missing	0	0.0	0	0.0	8	0.2	18	1.7	26	0.3
Sex										
Female	1234	49.8	662	50.7	1958	51.4	532	49.1	4386	50.5
Male	1244	50.2	645	49.3	1851	48.6	533	49.2	4273	49.2
Missing	0	0.0	0	0.0	1	0.02	18	1.7	19	0.3
Pregnancy^a^										
No	604	96.6	198	95.7	875	94.5	128	92.1	1805	95.2
Yes	21	3.4	6	2.9	50	5.4	3	2.2	80	4.2
Missing	0	0.0	3	1.4	1	0.1	8	5.8	12	0.6
BMI (kg/m^2^)										
Underweight < 18.5	136	5.5	68	5.2	97	2.6	28	2.9	329	3.8
Normal 18.5–24.9	1087	43.9	651	49.8	1657	43.5	372	38.2	3767	43.4
Overweight 25.0–29.9	778	31.4	399	30.5	1375	36.1	328	33.7	2880	33.2
Obese 30.0–34.9	295	12.0	122	9.3	499	13.1	157	16.1	1073	12.4
Very obese > 35	164	6.7	45	3.4	175	4.6	88	9.0	472	5.4
Mean (±SD)	25.4 ± 5.6		24.9 ± 4.8		26.0 ± 4.9		27.0 ± 5.7		25.8 ± 5.2	
Median (IQR)	25.0 6.0		24.2 5.5		25.4 6.0		26.1 7.0		25.0 6.3	
Range	14.0–66.0		12.7–58.8		13.2–74.1		13.9–56.5		12.7–74.1	
Missing	18	0.7	22	1.7	7	0.2	110	10.2	157	1.8
Primary residence within 30 days before ED admission										
Private home	2437	98.4	1273	97.4	3604	95.1	954	88.1	8268	95.3
Nursing home	22	0.9	18	1.4	103	2.7	71	6.7	214	2.5
Homeless	6	0.2	0	0.0	0	0.0	0	0.0	6	0.1
Shared accommodation/hostel	12	0.5	0	0.0	76	2.0	15	1.4	103	1.2
Other	1	0.04	16	1.2	0	0.0	22	2.1	39	0.5
Missing	0	0.0	0	0.0	27	0.7	21	1.9	48	0.6
Smoking history during the last 30 days										
No	1784	72.0	665	51.3	2514	66.0	820	75.1	5783	66.6
Yes	690	27.8	630	48.7	985	25.9	241	22.3	2546	29.3
Missing	4	0.2	12	0.9	311	8.1	22	2.0	349	4.0
Chronic disease										
No	799	32.2	453	34.7	1822	47.8	273	25.2	3347	38.6
Yes	1678	67.7	854	65.3	1988	52.2	761	70.3	5281	60.9
Missing	1	0.04	0	0.0	0	0.0	49	4.5	50	0.5
Influenza vaccination 2019–2020										
No	1713	69.1	869	66.5	2495	65.5	524	48.4	5601	64.5
Yes	709	28.6	437	33.4	1301	34.1	440	40.6	2887	33.3
Missing	56	2.3	1	0.1	14	0.4	119	11.0	190	2.2
≥ 65 years^b^										
No	348	39.5	278	44.8	470	34.2	203	18.7	1299	37.4
Yes	516	58.6	343	55.2	897	65.4	334	30.8	2090	60.0
Missing	16	1.8	0	0.0	5	0.4	71	6.6	92	2.6
Recent hospitalization < 30 days										
No	2319	93.6	1276	97.6	3662	96.1	910	84.0	8167	94.1
Yes	148	6.0	31	2.4	148	3.9	79	7.3	406	4.7
Missing	11	0.4	0	0.0	0	0.0	94	8.7	105	1.2

^a^ Only women in childbearing age (15–49): France: *n* = 625, Germany: *n* = 139, Italy: *n* = 207 and Spain: *n* = 926.

^b^ Elderly patients (≥ 65 years): France: *n* = 880, Germany: *n* = 608, Italy: *n* = 621 and Spain: *n* = 1372.

### 3.2 Description of Influenza Cases

Among 8600 patients with available data on RT‐PCR result, 494 (5.7%) tested positive for influenza, with the rate varying between countries: 1.7% (*n* = 18) in Germany, 4.0% (*n* = 52) in Italy, 4.9% (*n* = 121) in France and 7.9% (*n* = 303) in Spain (*p* < 0.001) (data not shown). Two‐thirds of influenza cases were of type A, and approximately 54% were A/H1N1(pdm09) (Table S6). For France, the proportion of influenza A and B were approximately similar (51% and 49%, respectively) compared to other countries who the main type was influenza A (Table S6). The proportion of influenza cases decreased with increasing age: 7.9% (*n* = 276) in the 18–49 years versus 5.3% (*n* = 88), 4.5% (*n* = 88) and 2.8% (*n* = 42) in the 50–64, 65–79 and ≥ 80 years, respectively (*p* < 0.001) (data not shown). The proportion of hospitalized patients was higher among influenza cases with type A than among influenza cases with type B (27.6% vs. 15.0%, respectively, *p* = 0.003) (data not shown). Among young patients (18–64 years), it was significantly higher among influenza cases with type A than among cases with type B (18.6% vs. 10.1%, respectively, *p* = 0.033) (data not shown). However, no difference was showed among elderly patients (46.6% vs. 41.7%, respectively, *p* = 0.662) (data not shown).

The rate of ED visits with confirmed influenza was 575 per 10,000 ED visits (data not shown). Among ED visits with confirmed influenza, 23% resulted in hospitalization (Table S7). The rate increased significantly with age: 13% in the 18–49 years, 22% in the 50–64 years, 38% in the 65–79 years and 64% in the ≥ 80 years (*p* < 0.001) (data not shown). The rate of hospitalization with confirmed influenza among all hospitalization related to ED visits was 47 per 1000 hospitalizations all ages combined (data not shown).

### 3.3 Description of ED Visits and ED‐Discharge Diagnoses

Among 8502 patients with available data on discharge diagnosis, almost 14% (*n* = 1179) had a respiratory discharge diagnosis, and only 2.2% (*n* = 183) had a discharge diagnosis coded as influenza (data not shown). The proportion with this diagnosis decreased with age: 4.1% (*n* = 141) in the 18–49 years versus 1.1% (*n* = 18), 1.0% (*n* = 19) and 0.3% (*n* = 5) in the 50–64, 65–79 and ≥ 80 years, respectively (data not shown). In contrast, the proportion of patients with a discharge diagnosis coded as pneumonia increased with age: 1.4% (*n* = 48) in the 18–49 years versus 2.2% (*n* = 37), 3.7% (*n* = 72) and 5.1% (*n* = 74) in the 50–64, 65–79 and ≥ 80 years, respectively (data not shown).

The rate of ED visits attributable to influenza as discharge diagnosis (i.e., Flu ICD) among all patients was 215 per 10,000 ED visits (data not shown). This is almost three times lower than the proportion of patients with LCI (5.8% or 575 per 10,000 ED visits) (data not shown). Among ED visits for influenza as discharge diagnosis, 10% resulted in hospitalization (data not shown). The rate was 42% for 65 years and over and 6% for 18–64 years (data not shown). The rate of hospitalization for influenza among all hospitalization related to ED visits was 8 per 1000 hospitalizations all ages combined (data not shown).

Among 490 influenza cases with available data on discharge diagnosis, 52% had a respiratory discharge diagnosis. Less than 25% were diagnosed as having influenza (Tables 2 and S7). It was higher in the age group 18–49 years than other age groups: 34% versus 14%, 12% and 10% in the 50–64, 65–79 and ≥ 80 years, respectively (*p* < 0.001) (Table 2). Nonrespiratory discharge diagnosis was also common and represented more than 50% of discharged diagnoses among patients older than 50 years (*p* = 0.026) (Table 2). The proportion of influenza cases who had their discharge diagnosis coded as circulatory system diseases increased with increasing age: 1.1% in 18–49 years, 3.4% in the 50–64 years, 6.9% in the 65–79 years and 14.6% in ≥ 80 years (*p* < 0.001) (Table 2).

**TABLE 2:** Description of discharge diagnoses groups among patients with laboratory‐confirmed influenza by age group and severity,^a^ 2019–2020 (*n* = 494).

	18–49 years		50–64 years		65–79 years		≥ 80 years		Total		Hospitalized		Nonhospitalized	
	*N* = 276	%	*N* = 88	%	*N* = 88	%	*N* = 42	%	*N* = 494	%	*N* = 115	%	*N* = 377	%
Respiratory outcomes	160	58.0	42	47.7	37	42.0	18	42.9	257	52.0	59	51.3	197	52.3
Acute upper respiratory infections	32	11.7	7	8.0	1	1.2	1	2.4	41	8.4	1	0.9	40	10.7
Influenza	93	33.9	12	13.6	10	11.5	4	9.8	119	24.3	16	13.9	102	27.3
Pneumonia	9	3.3	8	9.1	11	12.6	3	7.3	31	6.3	24	20.9	7	1.9
Other upper and lower respiratory tract diseases	11	4.0	3	3.4	4	4.6	2	4.9	20	4.1	3	2.6	17	4.6
Chronic respiratory diseases	4	1.5	2	2.3	5	5.8	2	4.9	13	2.7	5	4.4	8	2.1
Other respiratory diseases	11	4.0	10	11.4	6	6.9	6	14.6	33	6.7	10	8.7	23	6.2
Nonrespiratory outcomes	114	41.3	46	52.3	50	56.8	23	54.8	233	47.2	56	48.7	177	46.9
Infectious diseases	5	1.8	4	4.6	3	3.5	1	2.4	13	2.7	3	2.6	10	2.7
Neoplasms	0	0.0	0	0.0	1	1.2	0	0.0	1	0.2	1	0.9	0	0.0
Endocrine system diseases	2	0.7	0	0.0	1	1.2	1	2.4	4	0.8	3	2.6	1	0.3
Circulatory system diseases	3	1.1	3	3.4	6	6.9	6	14.6	18	3.7	7	6.1	11	2.9
Mental disorders	2	0.7	2	2.3	1	1.2	1	2.4	6	1.2	1	0.9	5	1.3
Nervous system diseases	10	3.7	4	4.6	5	5.8	0	0.0	19	3.9	5	4.4	14	3.7
Digestive system diseases	19	6.9	5	5.7	5	5.8	2	4.9	31	6.3	9	7.8	22	5.9
Genitourinary system diseases	6	2.2	4	4.6	3	3.5	2	4.9	15	3.1	6	5.2	9	2.4
Skin tissue diseases	3	1.1	0	0.0	0	0.0	0	0.0	3	0.6	1	0.9	2	0.5
Musculoskeletal and connective tissue diseases	3	1.1	2	2.3	1	1.2	0	0.0	6	1.2	0	0.0	6	1.6
Pregnancy, congenital malformations and other related	1	0.4	0	0.0	0	0.0	0	0.0	1	0.2	1	0.9	0	0.0
Various	60	21.9	22	25.0	24	27.6	10	24.4	116	23.7	19	16.5	97	25.9
Missing	2	0.7	0	0.0	1	1.2	1	2.4	4	0.8	0	0.0	3	0.8

^a^ A severe influenza was defined as patient with laboratory‐confirmed influenza who were hospitalized after leaving emergency department.

### 3.4 Accuracy of Coding for Influenza as Diagnostic When Leaving ED

Among patients with influenza ICD code–related discharge diagnosis, 65.0% were LCI. This correlation was higher among 18–64 years than ≥ 65 years but nonsignificant (66.0% vs. 58.3%, respectively, *p* = 0.461) (data not shown). The Se, Sp, PPV and NPV were 24.3%, 99.2%, 65.0% and 95.5%, respectively (Table 3). Se was different across age categories. It was higher among 18–49 years compared to other groups (33.9% in the 18–49 years vs. 13.6%, 11.5% and 9.8% in the 50–64, 65–79 and ≥ 80 years, respectively) (Table 3). Similarly, Se was different across the presence of at least one chronic disease and influenza vaccination status (Table 3).

**TABLE 3:** Accuracy of coding for influenza as diagnostic when leaving ED (i.e., Flu ICD codes) (*n* = 8442).

Variables	Percentage (95% CI)			
	Sensitivity	Specificity	Positive predictive value	Negative predictive value
Influenza discharge diagnosis	24.3 (23.4–25.2)	99.2 (99.0–99.4)	65.0 (64.0–66.0)	95.5 (95.1–96.0)
Age (years)				
18–49	33.9 (32.4–35.5)	98.5 (98.1–98.9)	66.0 (64.4–67.5)	94.5 (93.7–95.3)
50–64	13.6 (12.0–15.0)	99.6 (99.3–99.9)	66.7 (64.4–69.0)	95.3 (94.3–96.3)
65–79	11.5 (10.1–12.9)	99.5 (99.2–99.8)	52.6 (50.4–54.9)	95.9 (95.1–96.8)
≥ 80	9.8 (8.2–11.3)	99.9 (99.8–100)	80 (77.9–82.1)	97.4 (96.6–98.3)
Sex				
Female	23.2 (21.9–24.5)	99.2 (98.9–99.5)	64.4 (63.0–65.9)	95.4 (94.8–96.0)
Male	25.4 (24.1–26.7)	99.2 (98.9–99.5)	65.6 (64.2–37.0)	95.6 (95.0–96.2)
Chronic disease				
No	32.6 (31.0–34.3)	98.5 (98.0–98.9)	63.2 (61.5–64.9)	94.8 (94.0–95.5)
Yes	16.1 (15.1–17.1)	99.6 (99.5–99.8)	69.0 (67.7–70.2)	95.9 (95.4–96.5)
Influenza vaccination 2019–2020				
No	29.8 (28.6–31.1)	98.9 (98.6–99.2)	66.1 (64.8–67.3)	95.1 (94.5–95.6)
Yes	6.2 (5.3–7.1)	99.8 (99.7–100.0)	58.3 (56.5–60.2)	96.2 (95.5–96.9)

Abbreviation: CI: confidence interval.

### 3.5 Clinical Signs and Symptoms of Influenza Cases

Among 474 influenza cases with available data on symptoms, fever was the most frequent systemic symptom (62.0%) and cough, the most frequent respiratory symptom (67.7%) (data not shown). These symptoms were also the most frequent not only among 18–64 years (68.4% and 72.4%, respectively) but also among elderly (43.4% and 54.1%, respectively) (data not shown). Except for shortness of breath, all signs or symptoms decreased significantly when age increased (Figures S1 and S2).

In all, 23.0% were asymptomatic. The proportion of asymptomatic increased with age: 18% (*n* = 49) in 18–49 years, 21% (*n* = 18) in the 50–64 years, 33% (*n* = 27) in the 65–79 years and 37% (*n* = 15) in ≥ 80 years (*p* = 0.006) (Figure S3).

## 4 Discussion

The main objective of this multicentre prospective study was to measure the proportion of nontraumatic ED visits infected with influenza during the influenza season 2019–2020. We found that the proportion of patients with confirmed influenza was 5.8% (or 575 per 10,000 ED visits). This is almost three times higher than the proportion of patients with influenza diagnosis (2.2% or 215 per 10,000 ED visits) when leaving ED, and the ratio of both was statistically significant (*p* < 0.001).

Among 183 patients with influenza discharge ICD code–related diagnosis, 65.0% had an LCI. On the other hand, among the 494 LCI cases, only 24.3% had a discharged ICD code–related diagnosis of influenza. Few studies are similar to ours, with differences in the target population (paediatric patients) [11, 12] and also in the ICD codes used for influenza, which differ slightly [13, 14, 15]. In addition, the majority are retrospective studies using routine medical data and based on hospitalized patients due to missing data on the PCR test for influenza for outpatients. However, all concluded that diagnosis of influenza based on ICD influenza‐specific codes (i.e., ICD‐09 [487 or 488] or ICD‐10 [J09, J10 or J11]) underestimated the real burden of influenza among patients who visited ED. Moreover, coding of influenza may depend on the physicians and their clinical experience, medical training or coding practice in their unit. Consequently, some physicians may prefer to code the signs or symptoms rather than the disease as clinical diagnostic [16]. This can also be explained by the fact that not all patients presenting to ED with influenza symptoms are tested for the virus, particularly if they have no signs of severity or comorbidities and if the physician does not intend to admit them to hospital. There are a number of reasons why these patients are not routinely tested, such as the time required to obtain PCR results or of the nonavailability of rapid test in ED. In our study, we showed a lower Se (24.3%) and a higher Sp (99.2%) of coding for influenza compared to the study of Wade et al. who showed that the Se of coding for influenza was lower when test results were not available at discharge and the use of rapid PCR at ED was associated with greater coding accuracy for influenza [14]. In our study, all participants were sampled for the study in the ED, but we did not report whether any of them had or not a sample taken as part of their routine care when the physician suspected an infection by influenza. In our study, among influenza cases, the proportion of elderly patients with circulatory system diseases as clinical diagnosis was 9.4% versus 1.7% among 18–64 years. On the other hand, they were more often diagnosed for pneumonia (10.9% vs. 4.7% for 18–64 years). This can also be explained by the fact that older people have more comorbidities, and influenza could be the cause of a decompensation of a chronic disease such as a respiratory or cardiovascular disease and can progress to more severe pneumonia and so, influenza was not their main clinical diagnosis when leaving ED [13, 17].

The most frequent symptoms among influenza cases were cough (67.7%) and fever (62.0%) in this study. These symptoms were also reported as most common symptoms of influenza in other studies [16, 18, 19]. Symptoms being not specific to influenza, other respiratory viruses such as respiratory syncytial virus (RSV) that circulated as the same time as influenza can cause similar signs or symptoms [18, 20]. Testing patient at ED is therefore essential for controlling influenza not only during seasonal epidemics but also when other respiratory viruses as RSV or SARS‐CoV‐2 are circulating during the same period [21, 22], particularly if an asymptomatic patient needs to be hospitalized. In this way, the emergency physician can provide adequate care to the patient within a short time (e.g., the use of appropriate antivirals) and take steps to control the infection if necessary (i.e., to prevent possible nosocomial transmission). On the other hand, our study showed that signs or symptoms of influenza decreased when age increased and one elderly in three had no symptoms typically associated with influenza (34.4%). Several studies showed that older patients with LCI could have atypical clinical signs [23, 24, 25].

In our study, the overall rate of vaccination against influenza was 34.0% across all ages. It was 61.7% in elderly patients and 23.4% in at‐risk patients aged 18 to 64. The rate varied from country to country among elderly patients: 55% in Italy, versus 60%, 62% and 66% in France, Germany and Spain, respectively. They are below the WHO target of 75%. Although the effectiveness of the influenza vaccine varies according to influenza season and influenza subtype in circulation, vaccination remains one of the essential measures for preventing and controlling the disease. The introduction of the high‐dose influenza vaccine in these countries is an additional argument for encouraging the elderly to be vaccinated. It can help reduce the impact of influenza in terms of the number of cases, severity but also healthcare costs and quality of life [26, 27].

The main strengths of our study are its multicentre design, which increases the external validity of the results, and also the prospective collection of biological samples and clinical data. Potential classification bias was limited by the fact that all participating countries used the same standardized protocol and questionnaire and made similar virological tests. To our knowledge, this is the sole prospectively conducted multicentre study that has estimated the real burden of influenza within EDs, using PCR test as the gold standard for documenting confirmed cases of influenza.

Some limitations should also be mentioned. Firstly, the influenza ICD coded in this study was based only on the main discharge diagnosis. We therefore underestimated the proportion of patients with an influenza diagnosis by not considering secondary or associated diagnoses that may be coded as influenza. Secondly, the study was carried out during a single influenza season. Circulating strains may vary depending on the outbreak and the geographical location. In addition, the study was interrupted prematurely by the COVID‐19 pandemic at the beginning of March 2020 [28]. However, the period of the study included the peak epidemic [28, 29].

## 5 Conclusion

In conclusion, our study reinforces what other studies have shown before us: (1) Clinical diagnosis of influenza needs to be expanded to other ICD codes such as pneumonia or cardiovascular disease and (2) elderly patients can present with a clinical presentation of influenza that is not ILI as defined by ECDC or WHO, which used by many countries in Europe for influenza surveillance.

Our results corroborate the impact of influenza on patients visiting EDs in European countries, an impact that cannot easily be assessed on the basis of clinical presentation and medical records. Preventing influenza in the adult population can reduce healthcare use. In particular, it involves vaccinating people at risk, especially the elderly, who are the most vulnerable.

## Author Contributions

**Sélilah Amour:** conceptualization, methodology, investigation, writing – original draft, formal analysis, visualization, data curation, writing – review and editing, project administration. **Alberto Perez Rubio:** conceptualization, methodology, investigation, funding acquisition, writing – review and editing, supervision, resources, project administration. **Andrea Orsi:** conceptualization, investigation, funding acquisition, methodology, writing – review and editing, supervision, resources, project administration. **Michael Oppert:** conceptualization, methodology, investigation, funding acquisition, writing – review and editing, supervision, resources, project administration. **Micha Loebermann:** conceptualization, investigation, methodology, funding acquisition, writing – review and editing, supervision, resources, project administration. **Carlos del Pozo Vegas:** conceptualization, methodology, investigation, funding acquisition, writing – review and editing, supervision, resources, project administration. **Karim Tazarourte:** conceptualization, methodology, investigation, writing – review and editing, supervision, resources. **Marion Douplat:** conceptualization, methodology, investigation, writing – review and editing, supervision, resources. **Laurent Jacquin:** conceptualization, investigation, writing – review and editing, methodology, supervision, resources. **Giancarlo Icardi:** conceptualization, methodology, investigation, funding acquisition, writing – review and editing, supervision, resources. **Jonas Walker:** methodology, investigation, writing – review and editing. **Andrea Glass:** methodology, investigation, writing – review and editing. **Joshua Nealon:** conceptualization, methodology, writing – review and editing. **Sandra S. Chaves:** conceptualization, methodology, writing – review and editing. **FERS Study Group:** investigation. **Hélène Bricout:** conceptualization, methodology, funding acquisition, supervision, writing – review and editing. **Philippe Vanhems:** conceptualization, funding acquisition, writing – review and editing, methodology, data curation, supervision, resources, validation, investigation, writing – original draft, project administration.

## Conflicts of Interest

All authors have completed and submitted the ICMJE Form for the Disclosure of Potential Conflicts of Interest. J.N., S.S.C. and H.B. are Sanofi employees and may hold company share and/or stock options in the company. A.P.R., A.O., M.L., L.J., G.I. and P.V. declare financial interests/personal relationships that may be considered as potential competing interests. S.A., M.O., C.d.P.V., K.T., M.D., J.W. and A.G. declare no competing interests.

## Supporting information


**Table S1.** Swabbing and laboratory practices to be used for research influenza virus at each study site.
**Table S2.** Discharge diagnoses groups created according to the major ICD categories, with the corresponding ICD‐9 and ICD‐10 codes assigned to each group. Description of the diseases is provided according to the ICD‐10 codes.
**Table S3.** Site information, patient population and recruitment according to study site.
**Table S4.** Baseline characteristics according to age group, 2019–2020 (*n* = 8652).
**Table S5.** Baseline characteristics according to laboratory‐confirmed influenza status, 2019–2020 (*n* = 8600).
**Table S6.** Influenza result according to the country, 2019–2020 (*n* = 494).
**Table S7.** ED visits characteristics according to laboratory‐confirmed influenza status, 2019–2020 (*n* = 8600)*.*
**Figure S1.** Percentage of influenza cases with a systemic sign/symptom (fever, malaise, headache or myalgia/arthralgia) by age group, 2019–2020 (*N* = 490).
**Figure S2.** Percentage of influenza cases with a respiratory sign (cough, sore throat or shortness of breath) by age group, 2019–2020 (*N* = 490).
**Figure S3.** Percentage of asymptomatic influenza cases by age group, 2019–2020 (*N* = 490).

## Data Availability

The authors have nothing to report.
