# Supplementary material for: Influenza in Adults Seeking Care at Seven European Emergency Departments: A Prospective Active Surveillance During the 2019–2020 Influenza Season
Source: Influenza Other Respir Viruses. 2024 Nov 20;18(11):e70040. doi: 10.1111/irv.70040 (PMC11578834; doi:10.1111/irv.70040)
Supplement: Supplementary file 1 — Table S1. Swabbing and laboratory practices to be used for research influenza virus at each study site. Table S2. Discharge diagnoses groups created according to the major ICD categories, with the corresponding ICD‐9 and ICD‐10 codes assigned to each group. Description of the diseases is provided according to the ICD‐10 codes. Table S3. Site information, patient population and recruitment according to study site. Table S4. Baseline characteristics according to age group, 2019–2020 (n = 8652). Table S5. Baseline characteristics according to laboratory‐confirmed influenza status, 2019–2020 (n = 8600). Table S6. Influenza result according to the country, 2019–2020 (n = 494). Table S7. ED visits characteristics according to laboratory‐confirmed influenza status, 2019–2020 (n = 8600). Figure S1. Percentage of influenza cases with a systemic sign/symptom (fever, malaise, headache or myalgia/arthralgia) by age group, 2019–2020 (N = 490). Figure S2. Percentage of influenza cases with a respiratory sign (cough, sore throat or shortness of breath) by age group, 2019–2020 (N = 490). Figure S3. Percentage of asymptomatic influenza cases by age group, 2019–2020 (N = 490). [file IRV-18-e70040-s001.docx]

**Supplementary material**

**Table S1** – Swabbing and laboratory practices to be used for research influenza virus at each study site

**Table S2** – Discharge diagnoses groups created according to the major ICD categories, with the corresponding ICD-9 and ICD-10 codes assigned to each group. Description of the diseases is provided according to the ICD-10 codes

**Table S3** - Site information, Patient population and recruitment according to study site

**Table S4** - Baseline characteristics according to age group, 2019-2020 (n=8652)

**Table S5** – Baseline characteristics according to laboratory confirmed influenza status, 2019-2020 (n=8600)

**Table S6** - Influenza result according to the country,2019-2020 (n=494)

**Table S7** – ED visits characteristics according to laboratory confirmed influenza status, 2019-2020 (n=8600)

**Figure S1** - Percentage of influenza cases with a systemic sign/symptom (fever, malaise, headache or myalgia/arthralgia) by age group, 2019-2020 (N=490)

**Figure S2** - Percentage of influenza cases with a respiratory sign (cough, sore throat or shortness of breath) by age group, 2019-2020, (N=490)

**Figure S3** - Percentage of asymptomatic influenza cases by age group, 2019-2020, (N=490)

**Table S1** – Swabbing and laboratory practices to be used for research influenza virus at each study site

|  | France | Germany | Italy | Spain |
| --- | --- | --- | --- | --- |
| sample collection | At ED admission by ward nurse | During ED stay by dedicated study personnel | During ED stay by dedicated study personnel | At ED admission by ward nurse |
| Specimen collection (nasopharyngeal swab/nasal swab/throat swab) | nasopharyngeal (NP) swab | NP swab | Throat swabs (NP not accepted). Throat + nasal swab would be possible | nasopharyngeal (NP) swab |
| Laboratory assay used | RT-PCR from NP swab | RT-PCR from NP swab | RT-PCR from throat swab | RT-PCR from NP swab |
| Routine testing practices and how this will work with the study | PCR used often routinely  A second test will be conducted for the study | PCR used in symptomatic subjects;  A second test was conducted for the study | Very few tests routinely performed | Very few routine tests performed |
| Timelines for results turnaround for the study | At the end of the study– not part of clinical care | At the end of the study– not part of clinical care | Monthly – not part of clinical care (surveillance only) |  |
| Subtypage/lineage (if any)) | Yes (subtypage/lineage) | Yes (subtypage/lineage) | Yes (subtypage/lineage) |  |
| Local laboratory/national reference center | National Reference Center for Influenza (with *Institut Pasteur*) | Local laboratory (University of Rostock Medical Center, Department of Microbiology and Hygiene; Labor Potsdam – Klinikum Ernst von Bergmann) | Local laboratory (Ligurian Regional Reference Laboratory for Influenza) | National Reference Center for Influenza (Valladolid) |
| Other relevant information | - the sample of the study was vortexed and aliquoted.  - 2 samples (2 x 1ml) per patient  - Storage at - 80°C  Swabs also tested for COVID-19 | - each sample was vortexed and aliquoted  - 2 samples (2 x 1ml) per patient  - Storage at - 80°C  Swabs also tested for other respiratory pathogens at Rostock using Seegene Allplex Respiratory Panels^®^ (Respiratory syncytial virus A and B, Adenovirus, Enterovirus, Metapneumovirus, Parainfluenza virus 1-4, Bocavirus 1-4, Coronavirus 229E/NL63/OC43, COVID-19, Human rhinovirus, Bordetella parapertussis, B. pertussis, Chlamydophila pneumoniae, Haemophilus influenzae, Legionella pneumophila, Mycoplasma pneumoniae, Streptococcus pneumoniae) | Swabs tested also for other respiratory pathogens (Respiratory syncytial virus A and B, Adenovirus, Enterovirus, Metapneumovirus  Parainfluenza virus 1-2-3-4, Bocavirus 1/2/3/4, Coronavirus 229E-NL63-OC43, Human rhinovirus |  |

| Discharge diagnose | ICD-9^†^ | ICD-10^‡^ | Category of diagnosis (according to ICD-10) |  |
| --- | --- | --- | --- | --- |
| Respiratory outcomes | | | |  |
| Acute Upper Respiratory Infections | 460-466 | J00-J06 | Acute upper respiratory infections |  |
| Influenza | 487-488 | J09-J11 | Influenza |  |
| Pneumonia | 480-486 | J12-J18 | Pneumonia |  |
| Other Upper & Lower Respiratory Tract Diseases | 470-478 | J30-J39 | Other diseases of upper respiratory tract |  |
|  |  | J20-J22 | Other acute lower respiratory infections |  |
| Chronic Respiratory Diseases | 490-496 | J40-J47 | Chronic lower respiratory diseases |  |
| Other Respiratory Diseases | 500-508 | J60-J70 | Lung diseases due to external agents |  |
|  |  | J80-J84 | Other respiratory diseases principally affecting the interstitium |  |
|  |  | J85-J86 | Suppurative and necrotic conditions of the lower respiratory tract |  |
|  |  | J90-J94 | Other diseases of the pleura |  |
|  |  | J95 | Intraoperative and postprocedural complications and disorders of respiratory system, not elsewhere classified |  |
|  | 510-519 | J96-J99 | Other diseases of the respiratory system |  |
| Non-respiratory outcomes | | | | |
| Infectious Diseases | 001-139 | A00-B99 | Certain infectious and parasitic diseases | |
| Neoplasms | 140-239 | C00-D49 | Neoplasms | |
| Endocrine System Diseases | 240-279 | E00-E89 | Endocrine, nutritional and metabolic diseases | |
| Circulatory System Diseases | 280-289 | D50-D89 | Diseases of the blood and blood-forming organs and certain disorders involving the immune mechanism | |
|  | 390-459 | I00-I99 | Diseases of the circulatory system | |
| Mental Disorders | 290-319 | F01-F99 | Mental, Behavioural and Neurodevelopmental disorders | |
| Nervous System Diseases | 320-389 | G00-G99 | Diseases of the nervous system | |
|  |  | H00-H59 | Diseases of the eye and adnexa | |
|  |  | H60-H95 | Diseases of the ear and mastoid process | |
| Digestive System Diseases | 520-579 | K00-K95 | Diseases of the digestive system | |
| Genitourinary System Diseases | 580-629 | N00-N99 | Diseases of the genitourinary system | |
| Skin Tissue Diseases | 680-709 | L00-L99 | Diseases of the skin and subcutaneous tissue | |
| Musculoskeletal & Connective Tissue Diseases | 710-739 | M00-M99 | Diseases of the musculoskeletal system and connective tissue | |
| Pregnancy, Congenital Malformations & Other Related | 630-679 | O00-O9A | Pregnancy, childbirth and the puerperium | |
|  | 740-759 | Q00-Q99 | Congenital malformations, deformations and chromosomal abnormalities | |
|  | 760-779 | P00-P96 | Certain conditions originating in the perinatal period | |
| Various | 780-799 | R00-R99 | Symptoms, signs and abnormal clinical and laboratory findings, not elsewhere classified | |
|  | 800-999 | S00-T88 | Injury, poisoning and certain other consequences of external causes | |
|  | V01-V91 | V00-Y99 | External causes of morbidity | |
|  | E000-E999 | Z00-Z99 | Factors influencing health status and contact with health services | |

**Table S2** - Discharge diagnoses groups created according to the major ICD categories, with the corresponding ICD-9 and ICD-10 codes assigned to each group. Description of the diseases is provided according to the ICD-10 codes

*^†^ICD-09 used by Italy; ^‡^ICD-10 used by France, Spain and Germany*

**Table S3** - Site information, Patient population and recruitment according to study site

|  | France | Germany | Italy | Spain |
| --- | --- | --- | --- | --- |
| Sites information | | | | |
| Number of site(s) | 2 | 2 | 1 | 2 |
| Timeline | 24h (day/night 50/50) | Rostock: 08:00 – 20:00 hrs.  Postdam: Day-time | 6 am to 6 pm (60-65% of the population seen in this ED) | 24h/day – 7days/week |
| Number of visits | 200 p./day for each site | Rostock: ≈35-40 p./day – Potsdam: ≈110/day | 220p./day | 300p./day |
| Non-trauma subjects/week | 70% | Rostock = 96% - Postdam ≈70% | 78% | 80% |
| Recruitment practices | | | | |
| Study period | 13/01/2020-09/03/2020* | Rostock: 27/01/2020 – 19/03/2020*  Potsdam: 04/02/2020-23/03/2020 | January – March 2020 | January-March 2020 |
| Study start | According to the influenza activity  National surveillance (Santé Publique France) – January 2020 | According to the influenza activity  National surveillance (RKI, Berlin) – January 2020 | According to the influenza activity  National surveillance (Superior Institute of Health) – January 2020 | According to protocol (1 january) |
| Expected number of patients included/included patients | 3,000 expected/2,478 included | 1100 + 1848 (Potsdam) expected/483 + 775 (Potsdam) included* | 5,000 expected/1,307 included | 5,000 expected/5,000 included |
| Time of day of recruitment | 08:00 - 20:00 (recruitment possible before 08:00 or after 20:00) | Rostock & Potsdam : 08:00 – 20:00 hrs. | 06:00 – 18:00 or 07:00 – 19:00 | 24 hours/day |
| Number of days per week | 5 days/week (Monday to Friday) | 7 days/week | 5 days/week (Monday to Friday) | 3-4 days/week (alternating days) |
| Method of sampling | None | None: staff will recruit as many subjects as possible | None: staff will recruit as many subjects as possible | 1 each 3 |
| Invitation to join study | - Dedicated study personnel to approach eligible patients  - Physician explain the study and collect consent of patient | Dedicated study personnel to approach eligible patients and obtain informed consent | Dedicated study personnel to approach subjects | - Dedicated study personnel to approach eligible patients  - Physician explain the study and collect consent of patient |
| Inclusion / exclusion criteria | At ED admission by study nurse  Triage traumatic (exclued)/non-traumatic (eligible) | Subjects at ED with emergency labled as “internal medical”, able to give informed consent and age > 18 years  At ED admission by study nurse (Potsdam) | ICD codes to define “traumatic” patients  Severe “code red” patients will be rare (go directly to medical care) | At ED admission by study nurse  Triage traumatic/non-traumatic |
| Other information | *Study halted early due to Covid-19 pandemic | *Rostock: study halted early due to Covid-19 pandemic |  |  |

**Table S4** *-* Baseline characteristics according to age group, 2019-2020 (n=8652)

|  | **18-49 yrs N=3526** | **50-64 yrs N=1671** | | **65-79 yrs N=1966** | **>80 yrs**  **N=1489** |
| --- | --- | --- | --- | --- | --- |
| **Sex**, n (%) |  |  | |  |  |
| Female | 1897 (53.8) | 803 (48.1) | | 903 (45.9) | 778 (52.2) |
| Male | 1629 (46.2) | 867 (51.9) | | 1063 (54.1) | 711 (47.8) |
| **Pregnancy^†^**, n (%) |  |  | |  |  |
| No | 1805/1885 (95.8) | - | | - | - |
| Yes | 80/1885 (4.2) | - | | - | - |
| **BMI (kg/m²)** |  |  | |  |  |
| N | 3487 | 1641 | | 1937 | 1448 |
| Mean (±SD) | 25.1 (±5.3) | 26.7 (±5.3) | | 26.6 (±5.1) | 25.5 (±4.6) |
| Median (IQR) | 24.0 (6.1) | 26.0 (6.4) | | 26.0 (5.9) | 25.0 (5.8) |
| Range | 13.8-74.1 | 12.7-66.0 | | 13.7-59.0 | 14.6-47.9 |
| **BMI**, n (%) |  |  | |  |  |
| Underweight (<18.5) | 172 (4.9) | 52 (3.2) | | 39 (2.0) | 64 (4.4) |
| Normal (18.5-24.9) | 1796 (51.5) | 612 (37.3) | | 717 (37.0) | 639 (44.1) |
| Overweight (25.0-29.9) | 982 (28.2) | 597 (36.4) | | 774 (40.0) | 524 (36.2) |
| Obese (30.0-34.9) | 358 (10.3) | 257 (15.7) | | 288 (14.9) | 170 (11.7) |
| Very obese (>35) | 179 (5.1) | 123 (7.5) | | 119 (6.1) | 51 (3.5) |
| **Primary residence within 30 days** **before ED admission, n (%)** | | |  |  |  |
| Private home | 3371/3509 (96.1) | 1643/1666 (98.6) | | 1921/1964 (97.8) | 1326/1483 (89.4) |
| Nursing home | 35/3509 (1.0) | 7/1666 (0.4) | | 33/1964 (1.7) | 138/1483 (9.3) |
| Homeless | 5/3509 (0.1) | 1/1666 (0.1) | | 0/1964 (0.0) | 0/1483 (0.0) |
| Shared accommodation/hostel | 79/3509 (2.3) | 6/1666 (0.4) | | 5/1964 (0.3) | 13/1483 (0.9) |
| Other | 19/3509 (0.5) | 9/1666 (0.5) | | 5/1964 (0.3) | 6/1483 (0.4) |
| **Smoking history during the last 30 days, n (%)** | | |  |  |  |
| No | 2073/3389 (61.2) | 1035/1671 (64.9) | | 1434/1896 (75.6) | 1235/1442 (85.6) |
| Yes | 1316/3389 (38.8) | 561/1671 (35.1) | | 462/1896 (24.4) | 207/1442 (14.4) |
| **Chronic disease**, n (%) |  |  | |  |  |
| No | 2315/3516 (65.8) | 581/1667 (34.9) | | 322/1961 (16.4) | 125/1476 (8.5) |
| Yes | 1201/3516 (34.2) | 1086/1667 (65.1) | | 1639/1961 (83.6) | 1351/1476 (91.5) |
| **Influenza vaccination 2019-2020**, n (%) | | |  |  |  |
| No | 3085/3455 (89.3) | 1217/1644 (74.0) | | 861/1933 (44.5) | 432/1448 (29.8) |
| Yes | 370/3455 (10.7) | 427/1644 (26.0) | | 1072/1933 (55.5) | 1016/1448 (70.2) |
| **Recent hospitalisation (<30 days),** n (%) | | |  |  |  |
| No | 3418/3507(97.5) | 1583/1655 (95.7) | | 1811/1940 (93.3) | 1348/1463 (92.1) |
| Yes | 89/3507 (2.5) | 72/1655 (4.3) | | 129/1940 (6.7) | 115/1463 (7.9) |

**Table S5** – Baseline characteristics according to laboratory confirmed influenza status, 2019-2020 (n=8600)

|  | **Influenza positive N=494** | | **Influenza negative N=8106** | ***P*-value** |
| --- | --- | --- | --- | --- |
| **Age (years)** |  | |  |  |
| N | 494 | | 8098 |  |
| Mean (±SD) | 49.0 (±20.6) | | 56.5 (±21.6) |  |
| Median (IQR) | 46.2 (35.3) | | 57.3 (37.2) | <10^-4^ |
| Range | 18-96.3 | | 18-107.6 |  |
| **Age - n (%)** |  | |  |  |
| 18 - 49 yrs | 276 (55.9) | | 3235/8098 (40.0) | 0.000 |
| 50 – 64 yrs | 88 (17.8) | | 1571/8098 (19.4) |  |
| 65 - 79 yrs | 88 (17.8) | | 1857/8098 (22.9) |  |
| >80 yrs | 42 (8.5) | | 1435/8098 (17.7) |  |
| **Sex**, n (%) |  | |  |  |
| Female | 253 (51.2) | | 4103/8105 (50.6) | 0.798 |
| Male | 241 (48.8) | | 4002/8105 (49.4) |  |
| **Pregnancy^†^**, n (%) |  | |  |  |
| No | 139/143 (97.2) | | 1660/1735 (95.7) | 0.382 |
| Yes | 4/143 (2.8) | | 75/1735 (4.3) |  |
| **BMI (kg/m²)** |  | |  |  |
| N | 491 | | 7970 |  |
| Mean (±SD) | 25.7 (±5.2) | | 25.8 (±5.2) |  |
| Median (IQR) | 25.0 (6.3) | | 25.0 (6.3) | 0.587 |
| Range | 15-46.8 | | 12.7-74.1 |  |
| **BMI**, n (%) |  | |  |  |
| Underweight (<18.5) | 16/491 (3.3) | | 313/7970 (3.9) | 0.480 |
| Normal (18.5-24.9) | 229/491 (46.6) | | 3516/7970 (44.1) |  |
| Pre-obesity (25.0-29.9) | 160/491 (32.6) | | 2701/7970 (33.9) |  |
| Obese (30.0-34.9) | 54/491 (11.0) | | 1009/7970 (12.7) |  |
| Very obese (>35) | 32/491 (6.5) | | 431/7970 (5.4) |  |
| **Primary residence within 30 days before ED admission**, n (%) |  | |  |  |
| Private home | 473/492 (96.1) | | 7742/8079 (95.8) | 0.019 |
| Nursing home | 7/492 (1.4) | | 205/8079 (2.5) |  |
| Homeless | 0/492 (0.0) | | 6/8079 (0.1) |  |
| Shared accommodation/hostel | 12/492 (2.4) | | 90/8079 (1.1) |  |
| Other | 0/492 (0.0) | | 36/8079 (0.5) |  |
| **Smoking history during the last 30 days**, n (%) |  | |  |  |
| No | 296/490 (60.4) | | 5443/7780 (70.0) | 0.000 |
| Yes | 194/490 (39.6) | | 2337/7780 (30.0) |  |
| **Chronic disease**, n (%) |  | |  |  |
| No | 245/493 (49.7) | | 3085/8075 (38.2) | 0.000 |
| Yes | 248/493 (50.3) | | 4990/8075 (61.8) |  |
| **Influenza vaccination 2019-2020**, n (%) |  | |  |  |
| No | 373/488 (76.4) | | 5196/7942 (65.4) | 0.000 |
| Yes | 115/488 (23.6) | | 2746/7942 (34.6) |  |
| **Recent hospitalisation (<30 days),** n (%) | |  |  |  |
| No | 481/491 (98.0) | | 7649/8045 (95.1) | 0.004 |
| Yes | 10/491 (2.0) | | 396/8045 (4.9) |  |

**Table S6** - Influenza result according to the country,2019-2020 (n=494)

|  | **France** | | **Italy** | | **Spain** | | **Germany** | | **Total** | |
| --- | --- | --- | --- | --- | --- | --- | --- | --- | --- | --- |
|  | **N =121** | **%** | **N=52** | **%** | **N=303** | **%** | **N=18** | **%** | **N=494** | **%** |
| **Influenza strain** | | | | | | | | | | |
| Influenza A | 62 | 51.2 | 38 | 73.1 | 207 | 68.3 | 18 | 100.0 | 325 | 65.8 |
| Influenza B | 59 | 48.8 | 14 | 26.9 | 80 | 26.4 | 0 | 0.0 | 153 | 31.0 |
| Unknown | 0 | 0.0 | 0 | 0.0 | 16 | 5.3 | 0 | 0.0 | 16 | 3.2 |
| **Influenza subtyped** | | | | | | | | | | |
| A/H1N1pdm09 | 51 | 42.2 | 25 | 48.1 | 19 | 59.1 | 12 | 66.7 | 267 | 54.1 |
| A/H3N2 | 10 | 8.3 | 12 | 23.1 | 4 | 1.3 | 4 | 22.2 | 30 | 6.1 |
| A not subtyped | 1 | 0.8 | 1 | 1.9 | 24 | 7.9 | 2 | 11.1 | 28 | 5.7 |
| B not subtyped | 4 | 3.3 | 10 | 19.2 | 47 | 15.5 | 0 | 0.0 | 61 | 12.4 |
| B/Victoria lineage | 53 | 43.8 | 4 | 7.7 | 33 | 10.9 | 0 | 0.0 | 90 | 18.2 |
| B/Yamagata lineage | 2 | 1.7 | 0 | 0.0 | 0 | 0.0 | 0 | 0.0 | 2 | 0.4 |
| Unknown | 0 | 0.0 | 0 | 0.0 | 16 | 5.3 | 0 | 0.0 | 16 | 3.2 |

**Table S7** – ED visits characteristics according to laboratory confirmed influenza status, 2019-2020 (n=8600)

|  | **Influenza positive N=494** | **Influenza negative N=8106** | ***P*-value** |
| --- | --- | --- | --- |
| **Length of stay in ER (hours)** |  |  |  |
| N | N=493 | N=8032 |  |
| Mean (±SD) | 6.2 (±8.7) | 6.3 (±7.9) |  |
| Median (IQR) | 4.0 (3.5) | 4.3 (4.0) | 0.002 |
| Range | 0.0-75.0 | 0.0-174.0 |  |
| **Discharge diagnosis,** n (%) |  |  |  |
| Respiratory outcomes | 257/490 (52.4) | 917/7952 (11.5) | 0.000 |
| Acute Upper respiratory infections | 41/490 (8.4) | 252/7952 (3.2) |  |
| Influenza | 119/490 (24.3) | 64/7952 (0.8) |  |
| Pneumonia | 31/490 (6.3) | 200/7952 (2.5) |  |
| Other upper & lower respiratory tract Diseases | 20/490 (4.1) | 110/7952 (1.4) |  |
| Chronic respiratory Diseases | 13/490 (2.7) | 128/7952 (1.6) |  |
| Other respiratory Diseases | 33/490 (6.7) | 163/7952 (2.1) |  |
| Non-respiratory outcomes | 233/490 (47.6) | 7035/7952 (88.5) |  |
| Infectious diseases | 13/490 (2.7) | 219/7952 (2.8) |  |
| Neoplasms | 1/490 (0.2) | 67/7952 (0.8) |  |
| Endocrine System Diseases | 4/490 (0.8) | 122/7952 (1.5) |  |
| Circulatory System Diseases | 18/490 (3.7) | 835/7952 (10.5) |  |
| Mental Disorders | 6/490 (1.2) | 197/7952 (2.5) |  |
| Nervous System Diseases | 19/490 (3.9) | 579/7952 (7.3) |  |
| Digestive System Diseases | 31/490 (6.3) | 919/7952 (11.6) |  |
| Genitourinary System Diseases | 15/490 (3.1) | 588/7952 (7.4) |  |
| Skin Tissue Diseases | 3/490 (0.6) | 197/7952 (2.5) |  |
| Musculoskeletal & Connective Tissue Diseases | 6/490 (1.2) | 450/7952 (5.7) |  |
| Pregnancy, Congenital Malformations & Other Related | 1/490 (0.2) | 32/7952 (0.4) |  |
| Various | 116/490 (23.7) | 2830/7952 (35.6) |  |
| **Emergency room outcome,** n (%) |  |  |  |
| Hospitalized | 113/492 (23.0) | 2284/8011 (28.5) | 0.040 |
| ICU admission (and hospitalisation) | 2/492 (0.4) | 62/8011 (0.8) |  |
| Discharged | 377/492 (76.6) | 5663/8011 (70.7) |  |
| Death | 0/492 (0.0) | 2/8011 (0.02) |  |
| **Length of stay in hospital (days)** |  |  |  |
| N | N=103 | N=2215 |  |
| Mean (±SD) | 8.6 (±8.3) | 8.0 (±8.7) |  |
| Median (IQR) | 6.0 (7.0) | 5.0 (7.0) | 0.306 |
| Range | 1.0-42.0 | 0.0-105.0 |  |
| **Final hospital outcome within 4 weeks after admission,** n (%) |  |  |  |
| Discharged to community | 91/105 (86.7) | 1772/2195 (80.7) | 0.209 |
| Discharged to long-term facility | 2/105 (1.9) | 81/2195 (3.7) |  |
| Discharged to other hospital/treatment facility | 8/105 (7.6) | 247/2195 (11.3) |  |
| Not discharged after 4 weeks | 3/105 (2.9) | 28/2195 (1.3) |  |
| Death | 1/105 (1.0) | 67/2195 (3.1) |  |

**Figure S1 –** Percentage of influenza cases with a systemic sign/symptom (fever, malaise, headache or myalgia/arthralgia) by age group, 2019-2020 (N=490). Number within bars represents the total population in each category

**Figure S2 –** Percentage of influenza cases with a respiratory sign (cough, sore throat or shortness of breath) by age group, 2019-2020, (N=490).

Number within bars represents the total population in each category

**Figure S3** – Percentage of asymptomatic influenza cases by age group, 2019-2020, (N=490).

Number within bars represents the total population in each category
